# Colossal heating efficiency via eddy currents in amorphous microwires with nearly zero magnetostriction

**DOI:** 10.1038/s41598-020-57434-8

**Published:** 2020-01-17

**Authors:** Irene Morales, Diego Archilla, Patricia de la Presa, Antonio Hernando, Pilar Marin

**Affiliations:** 1 Instituto de Magnetismo Aplicado, UCM-ADIF-CSIC, A6 22,500 Km, 20230 Las Rozas, Spain; 2 Departamento Física de Materiales, University Complutense de Madrid, 28040 Madrid, Spain; 3 Donostia International Physics Center DIPC, Paseo Manuel de Lardizabal 4, 2018 Donostia-San Sebastián, Spain and IMDEA Nanociencia, Faraday, 9, 28049 Madrid, Spain

**Keywords:** Materials science, Condensed-matter physics, Magnetic properties and materials

## Abstract

It is well stablished that heating efficiency of magnetic nanoparticles under radiofrequency fields is due to the hysteresis power losses. In the case of microwires (MWs), it is not clear at all since they undergo non-coherent reversal mechanisms that decrease the coercive field and, consequently, the heating efficiency should be much smaller than the nanoparticles. However, colossal heating efficiency has been observed in MWs with values ranging from 1000 to 2800 W/g, depending on length and number of microwires, at field as low as *H* = 36 Oe at *f* = 625 kHz. It is inferred that this colossal heating is due to the Joule effect originated by the eddy currents induced by the induction field B = M + χH parallel to longitudinal axis. This effect is observed in MWs with nearly zero magnetostrictive constant as Fe_2.25_Co_72.75_Si_10_B_15_ of 30 μm magnetic diameter and 5 mm length, a length for which the inner core domain of the MWs becomes axial. This colossal heating is reached with only 24 W of power supplied making these MWs very promising for inductive heating applications at a very low energy cost.

## Introduction

Inductive heating of ferromagnetic materials and composites is known as a renewed topic in different research fields with many applications among which hyperthermia cancer treatment stands out^1–3^. However, there is currently a growing interest in applications to which this concept can be spread out, such as heterogeneous catalysis^4–6^, electrolysis of water^7^, new methods of organic synthesis^8,9^, molecularly imprinted polymers^10^, and others^11,12^.

These new applications of the use of magnetic nanoparticles as nano-heaters activated by radiofrequency fields are being recently explored and open a new and wide range of possibilities in this field. First, unlike biological applications, biocompatibility is no longer a requirement, therefore, any type of metal, alloy or any magnetic oxide are of interest for this study. Second, the dispersion medium can be organic or inorganic, or even solid matrices. Finally, new frequency and amplitude field ranges can be explored because there is no biological limitation in the applied fields. The determining factor for these types of processes is that the energy cost has to be much smaller than the regarding current processes, either because the application of the radiofrequency field allows more effective processes or because the required temperature or pressure are reduced by the inductive heating^7,9,10^.

One of the fundamental requirements that a material should meet to make these new applications really interesting is that the energy needed to activate these nano-heaters is low, i.e., field amplitude and frequency has to be as low as possible. Only in this way, really efficient processes can be obtained.

The heating losses of magnetic materials can be originated by hysteresis losses and/or eddy currents^13^. Hysteresis losses are related to the magnetic domain and, consequently, to the hysteresis loop area of the material^14^. On the other hand, eddy-currents are generated by electromagnetic field induction and lead to Joule heating of the material. In most of the magnetic nanoparticle, the main heating mechanism is by hysteresis losses whereas the Joule effect can be discarded due the small size of the particles. However, if magnetic materials have the proper size and conductivity, this effect should also be considered.

The amorphous soft magnetic microwires (MWs) are one of the most studied soft magnetic system^15^, they are composed by a metallic core and a Pyrex shell. The magnetic behavior is given by the amorphous core but also by the stress induced by the shell, which acts as protection agains corrotion as well^16^. Depending on the quenching rates, a complex radial distribution of internal stresses with axial, radial, and circular component can appear. The main source of magnetic anisotropy is given by the interactions between induced mechanical stresses with local magnetic moments.

These MWs have extraordinary magnetic properties (e.g. magnetic bistability, giant magneto-impedance effect (GMI)^17,18^, magnetoelastic^19^ and ferromagnetic resonance^20^, etc) and have been used in a wide range of technological applications as wireless magnetic sensors^20^, microelectronics, security, biomedical applications^21^ and magnetic hyperthermia^14^.

The easy axis of magnetization is determined by magnetostriction constant. In positive magnetostrictive MWs (Fe-rich MWs), the inner core has an easy magnetization direction along the axis and the outershell easy direction points radially to the axis^22^. In the negative magnetostrictive MWs (Co-rich MWs), the inner-core easy magnetization is perpendicular to the axis direction while the external shell adopts circular directions^23^. On the other hand, nearly-zero magnetostrictive MWs have a singular domain structure: (1) the inner core can show either transverse or axial anisotropy, depending on the MWs dimensions (radius of the metallic nucleus and glass coating thickness); (2) the outer shell displays a circular easy axis with consecutive rings magnetized in opposite directions, the so-called bamboo-like outer domain structure^24^. The magnetic behavior is determined by this type of domain structure. Positive magnetostrictive MWs show a square hysteresis loop with large Barkhausen jump whereas those having nearly-zero magnetostrictive constant λ_s_ shows smaller susceptibility but in a higher field range because the circumferentially magnetized rings.

The heating efficiency of Fe-rich MWs with positive λ_s_ has been reported as a function of the field amplitude and the wire numbers^14^. Specific Loss Power (SLP) as high as 1000 W/g can be reached with 700 Oe and 330 kHz, depending on the MWs lengths and number. This SLP is surprisingly high since non-coherent demagnetisation processes should lead to smaller heating capabilities^25^. Therefore, the eddy-currents could be the origin of the heating in MWs, although, in principle, it has been reported that in the case of soft ferromagnetic glass-coated MWs, the eddy-current damping is often negligible due to the amorphous nature of the MWs presenting high electrical resistivity^26,27^. Evidently, the heating capacity of MWs deserves further investigations in order to understand the heating mechanisms under radiofrequency fields.

The present work shows the heating efficiency for nearly zero magnetrostictive Co-rich MWs which is investigated as a function of MWs length (*L*) (from *L* = 2.5 to 80 mm) and number (*n*) of MWs (from *n* = 1 to 20) subjected to radiofrequency fields ranging from 100 to 625 kHz and from 3 to 120 Oe. The magnetic domain structure seems to change from radial to longitudinal configuration as length decreases, and it is accompanied by an enhancing heating efficiency. This allows drawing the inference that the heating mechanism is mainly given by the contribution of eddy-currents, although a minor hysteresis losses contribution cannot be discarded at all. A colossal heating efficiency with *SLP* ≈ 2800 W/g is observed at *f* = 615 kHz and field amplitude as small as *H* = 36 Oe, with a total power supplied of only 24 W. Some experiments made in air shows that a unique MW with *L* = 5 mm subjected to *H* = 12 Oe at *f* = 625 kHz can increase its temperature by 5 °C in 5 s, with a power supplied of only 2 W. All these characteristics make the Co-reach MWs a promising material for induction heating applications. Just to remark the relevance of this result, it is worth noting that in the case of magnetic nanoparticles, one of the highest *SLP* value reported in the literature is ≈2300 W/g at *H* = 300 Oe and *f* = 700 kHz^28^, a field ten times higher than the value reported here which implies a much higher energy cost. Most of the magnetic nanoparticles with *SLP* values higher than 1000 W/g require fields higher than 300 Oe with the consequent energy cost^29–31^.

## Results

### AC-hysteresis loops at 50 Hz

**Figure 1:**
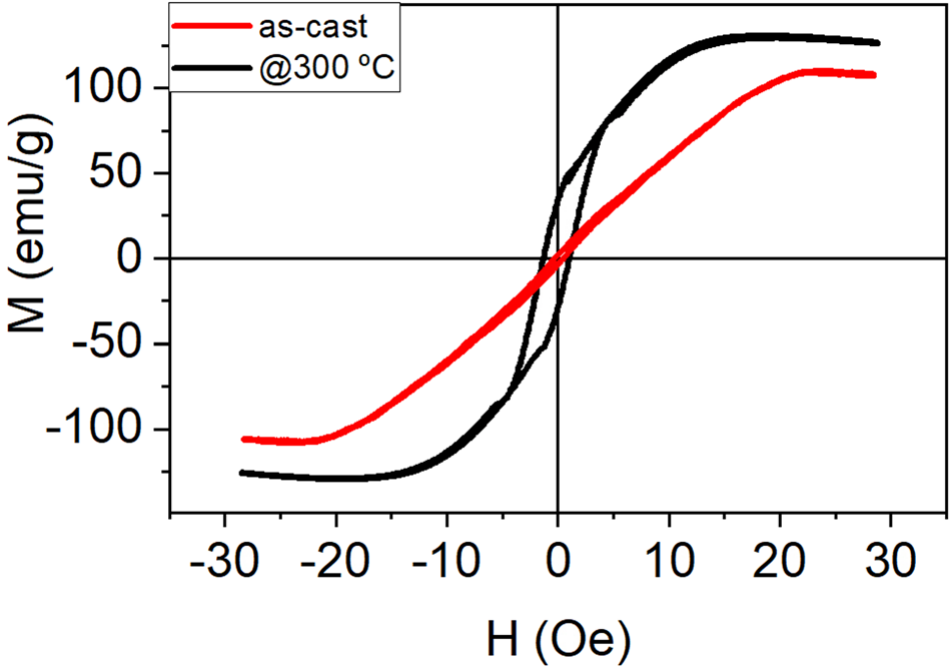
Hysteresis loops at room temperature of MW30 with *L* = 80 mm measured as-cast and annealed at 300 °C.

A conventional 50 Hz induction method was used to characterize axial hysteresis loops of the MWs. The hysteresis loops for as-cast and annealed samples of MW30 (a MW with magnetic diameter *d*_*m*_ = 31.4 μm) are shown in Fig. 1 for a single MW with *L* = 80 mm. The magnetic behaviour of the MWs depends strongly on the domain structure and is determined by minimization of magnetoelastic energy ${K}_{me}=3/2{\lambda }_{s}{\sigma }_{ii}$, where *λ*_*s*_ refers to saturation magnetostriction constant and σ_ii_ is the dominant internal stress component.

**Figure 2:**
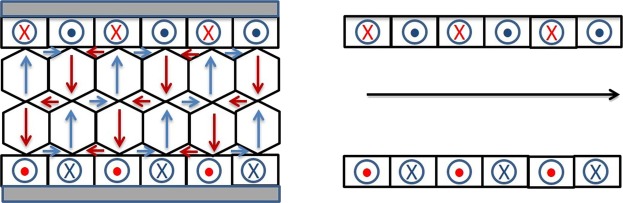
Magnetic domains for amorphous glass-coated MWs with almost zero magnetostriction (left) and magnetic domains after glass removing (see ref. ^16^).

MW30 with *L* = 80 mm shows saturation magnetization *M*_*s*_ = 107 emu/g, saturation field *H*_*s*_ = 22 Oe and coercivity *H*_*c*_ = 0.4 Oe, whereas remanence *M*_*r*_ is almost negligible. When the sample is annealed, *M*_*s*_ and *H*_*c*_ increase up to 130 emu/g and 1.2 Oe, respectively, but *H*_*s*_ decreases down to 12 Oe (see Fig. 1). Both hysteresis curves show a negative slope at high fields due to the diamagnetic contribution of the glass coating. In this type of MWs the calculated radial distribution of internal stresses in combination with negative *λ*_*s*_ gives a domain structure with a radially magnetized inner and a circumferentially magnetized outer shell. The radial magnetization from inner core would lead to severe magnetostatic and exchange interactions so that the magnetization in this region has also small axial component (see Fig. 2).

A decrease in the mechanical stress entails an increase in susceptibility accompanied by a decrease in the anisotropy field due to negative λ_s_. In this structure, the magnetostatic and exchange terms increase substantially and, after glass removal, the easy axis of inner core changes from radial to axial and the circumferential anisotropy of the outer shell becomes stronger. The volume of the outer shell increases at expense of the inner core^16^.

The thermal treatment of MWs helps to release internal stresses of the wires and this can modify the susceptibility, the magnetic anisotropy and the electrical resistivity, as well^32–34^. Therefore, the susceptibilities increase for the annealed samples should be given mainly by the stress release due to the thermal treatment^35,36^.

**Figure 3:**
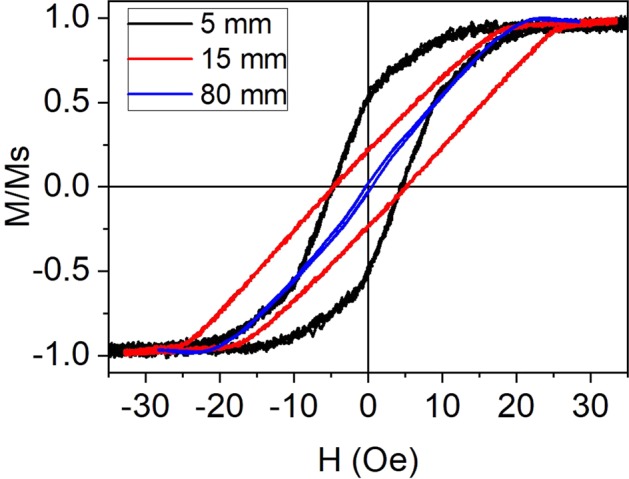
Hysteresis loops of a single MW30 with *L* = 5, 15 and 80 mm.

In the case of MW30, the magnetic behaviour for lengths shorter than 80 mm is quite different. Figure 3 shows the hysteresis loops of a single MW30 with three different lengths: *L* = 5, 15 and 80 mm. As can be seen, the shorter the MWs, the higher the remanence and coercivity are. *H*_*C*_ and *M*_*r*_ are almost negligible for *L* = 80 mm whereas for *L* = 15 and 5 mm, the coercivity increases to 5 Oe and *M*_*r*_ increases up to 20 and 50%, respectively. It is known that the length affects the magnetization reversal process in soft amorphous MWs^37^. The magnetic behaviour of the longest MW30 corresponds to the behaviour of low negative magnetostrictive MWs^32,38^, whereas the susceptibility increases as MW’s length is reduced. As previously commented, the relative permeability of amorphous MWs with nearly zero magnetostriction strongly depends on the external stress. The highest value of the relative permeability is obtained for samples with no applied stress, and the glass-coating is the actuator of this stress on the amorphous MWs^16^. The demagnetizing energy is almost negligible because demagnetizing factors are smaller than 10^−5^ for length to diameter ratios higher than 200, as in the case of the shortest length *L* = 5 mm^39^. Therefore, the anisotropy effectively controls and determines the magnetic properties of these MWs, even for short lengths. These results suggest that length reduction makes the magnetic anisotropy changes from radial to axial, as in the case of the MWs with removed glass cover (see Fig. 2).

**Figure 4:**
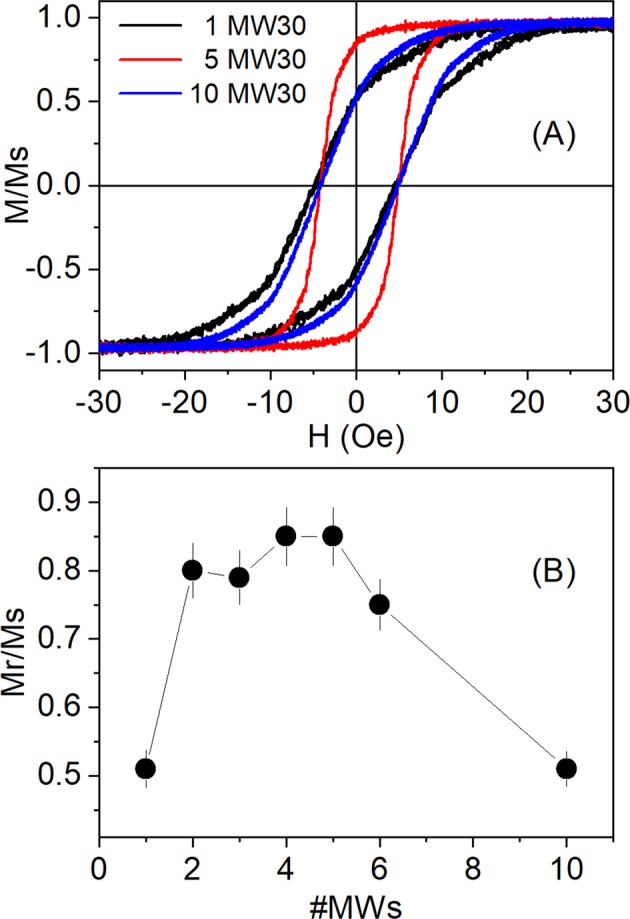
(**A**) Hysteresis loops for 1, 5 and 10 MW30 with *L* = 5 mm. (**B**) Mr/Ms as a function of the number of MW30 with *L* = 5 mm.

**Figure 5:**
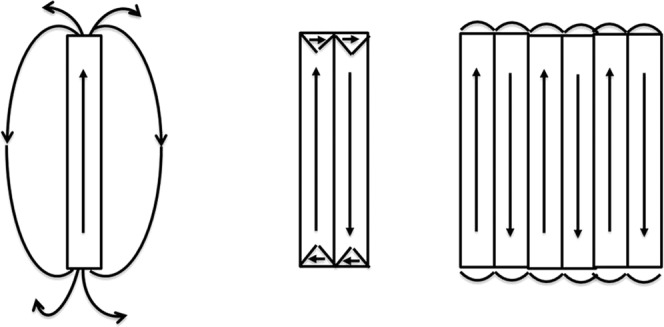
Domain structures for different number of MW30 with *L* = 5 mm. Left: a single MW. Center: two MWs with closure domain. Right: structure domain of a large number of MWs.

Once the magnetic behaviour of short MWs has been established, it is interesting to know which is the role of magnetostatic interactions when several short MWs are put side by side. The previous works report that the magnetic behaviour can change dramatically when 1 or 2 MWs are put side by side as a consequence of the magnetostatic interactions^40–42^. As previously established, the short length of the MWs induces a domain structure with the magnetization along the MWs. However, when 2 MWs are put together, the remanence increases from *M*_*r*_ ≈ 50% for *n* = 1 up to *M*_*r*_ ≈ 80% for *n* = 2, suggesting the formation of closure domains in order to diminish the magnetostatic energy. From Fig. 4, it is observed that this *M*_*r*_ is preserved up to 6 MWs, then, it decreases again to *M*_*r*_ = 50% for 10 MWs. These results suggest that domain structure changes for a high number of MWs, in order to decrease the magnetostatic energy. A possible domain structure for different number of MWs that could explain this behaviour is shown in Fig. 5.

### Calorimetry at radiofrequency fields

The heating efficiency of MWs has been investigated under two different conditions: (a) in water and (b) in air. (a) In the first case, 20 MWs have been set in the tip of a micropipette with the opposite site sealed and filled with 0.5 ml distillated water. It is important to have all the MWs parallel to the applied field. (b) The MWs were pricked in a base of polystyrene.

The heating efficiency of the magnetic materials is measured from the temperature increase *ΔT* of a given mass of the MWs in a determined mass of fluid *m*_*F*_ during the time interval *Δt* of the experiment. The expression for the specific loss power of the magnetic material is given by^43^:

1 $$SLP=\frac{{m}_{F}{C}_{F}+{m}_{MW}{C}_{M{W}_{s}}}{{m}_{MW}}\frac{\Delta T}{\Delta t}$$

where *C*_*F*_ and *C*_*MWs*_ are the specific heat capacities of the fluid (water or air) and the MWs, respectively.

The methodology for each measurement has been as follow: Prior to turning the magnetic field on, the sample temperature has been recorded for about 30 s to ensure thermal stability and to have a baseline for the calculation of the *SLP*. When the field has been turned on, the temperature increase has been measured either during 300 s or up to 80 °C for MWs in water, well below the corresponding boiling temperatures. In the case of the measurements in air, the field has been turned off at 100 °C.

#### MWs in water

In the case of MWs in water, the largest magnetic mass of MWs measured in these experiments was 0.54 mg in a total mass of 500 mg of water, giving a MW concentration smaller than 1%. Therefore, the low mass concentration allows discarding the specific heat capacity of the MWs and the SLP can be calculated as:

2 $$SLP=\frac{{C}_{water}}{{m}_{MW}}\frac{\Delta {T}}{\Delta t}$$

where *C*_*water*_ is the specific heat capacity of water (4.185 J/(g K)).

**Figure 6:**
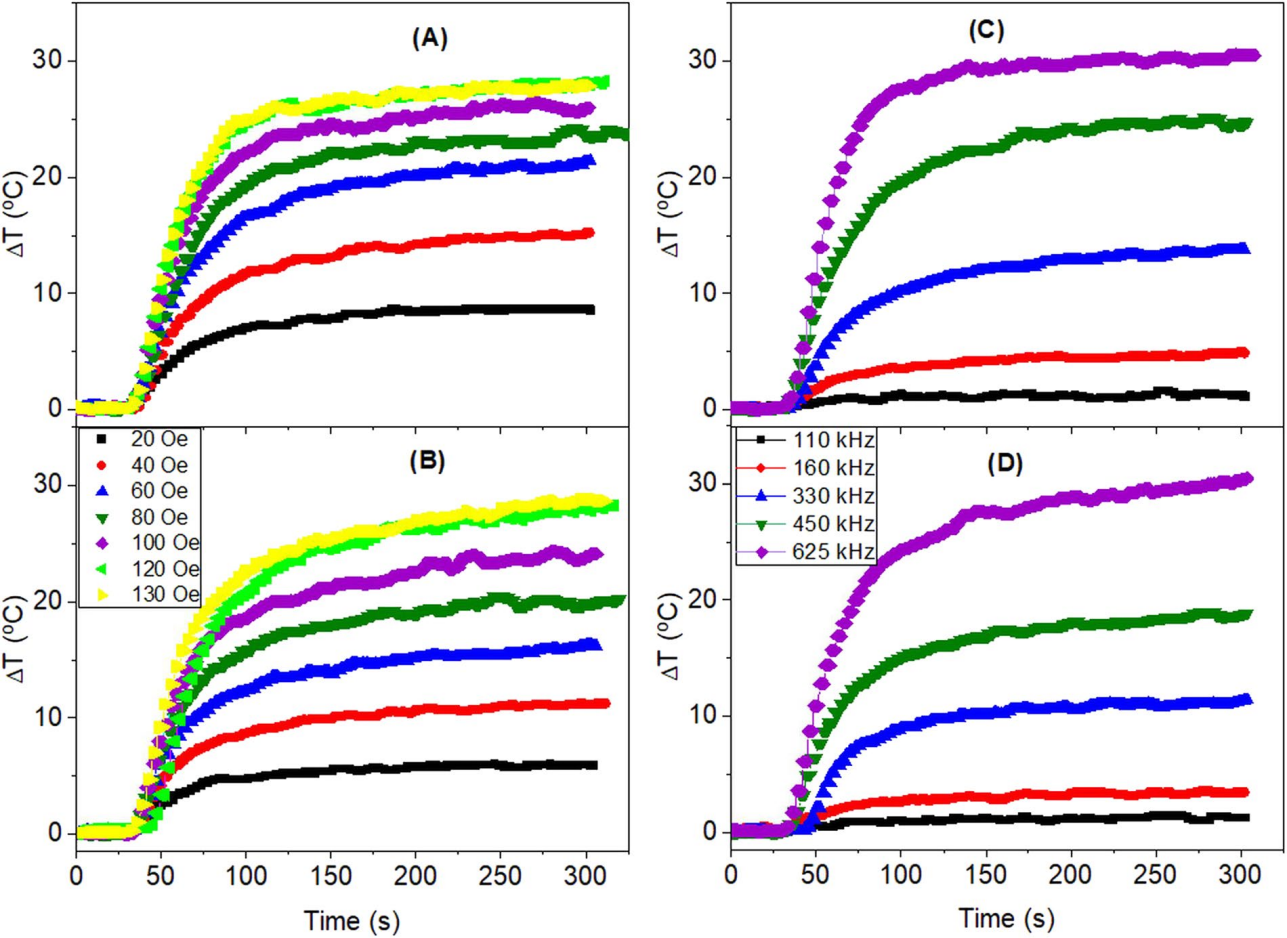
Temperature increase of the as-cast (**A**,**C**) and annealed at 300 C (**B**,**D**) MW30 as a function of field amplitude with field frequency *f* = 331 kHz (**A**,**B**) and as a function of field frequency at *H* = 36 Oe (**C**,**D**). (*n* = 20, *L* = 5 mm).

**Figure 7:**
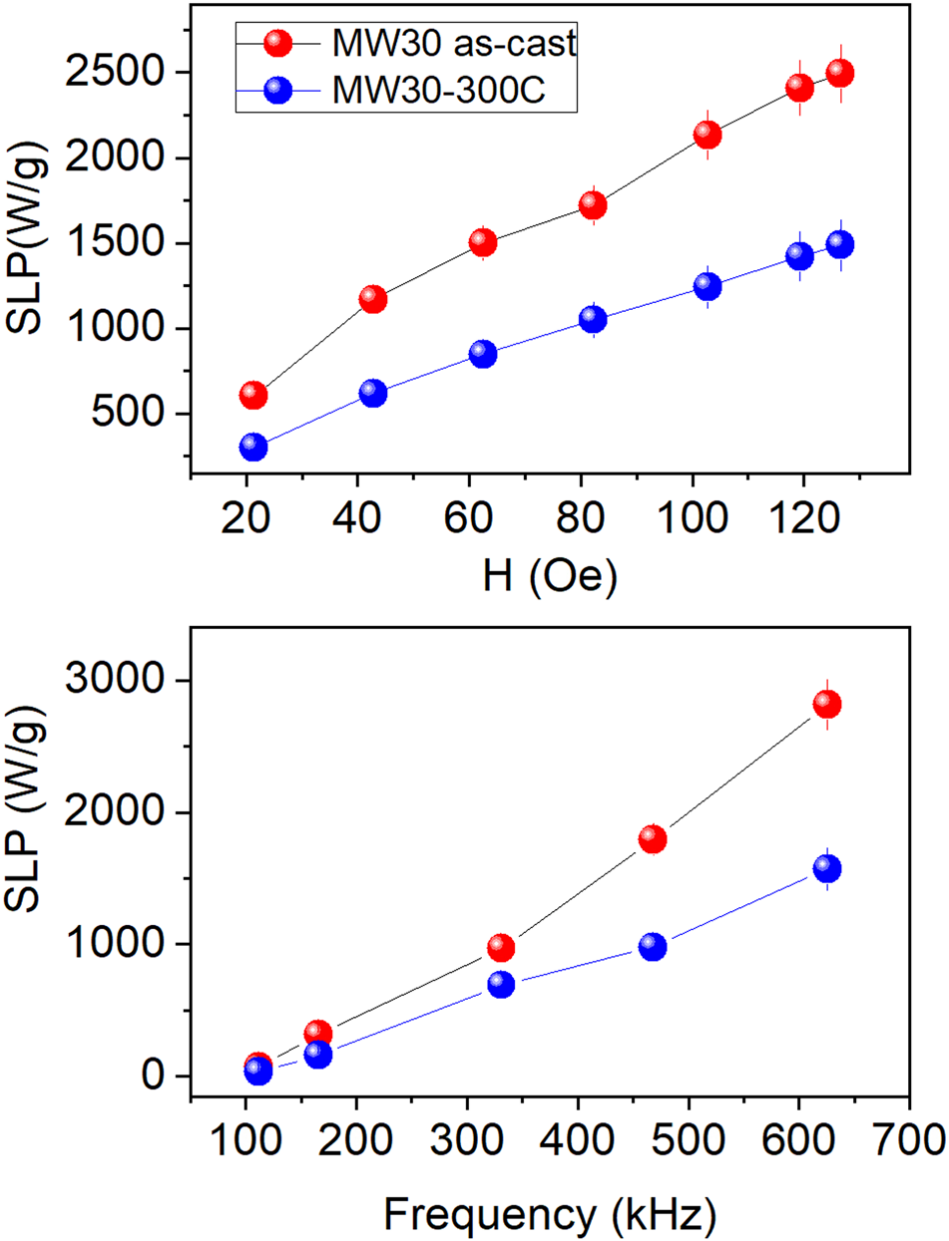
SLP as a function of field amplitude at *f* = 331 kHz (above) and as a function of frequency at *H* = 36 Oe (below) for as-cast MW30 (red circles) and annealed at 300 C (blue circles). The SLP is calculated per unit of magnetic mass. (*n* = 20, *L* = 5 mm).

The heating curves of as-cast and annealed MW30 have been measured in water for 20 MWs with *L* = 5 mm as a function of field amplitude at *f* = 331 kHz and of field frequency at *H* = 36 Oe. The Figs. 6 and 7 show the heating curves and the *SLP* for as-cast and thermal annealed MWs calculated with Eq. 1.

The results show all MWs have a huge heating efficiency since they have *SLP* ∼ 600 W/g, even at fields as low as 20 Oe and it increases up to 2500 W/g at 120 Oe, which is still a relatively low field. No quadratic dependence with the field is observed. For *H* = 36 Oe, the frequency dependence of both samples shows a similar *SLP* at low frequencies, whereas at higher frequencies (*f* > 330 kHz), the as-cast MWs show a heating efficiency much higher than the annealed ones. It is worth noting the colossal *SLP* value, which is around 2800 W/g, by applying only 36 Oe at 625 kHz. In the following experiments, all results are focused on the as-cast MWs.

**Figure 8:**
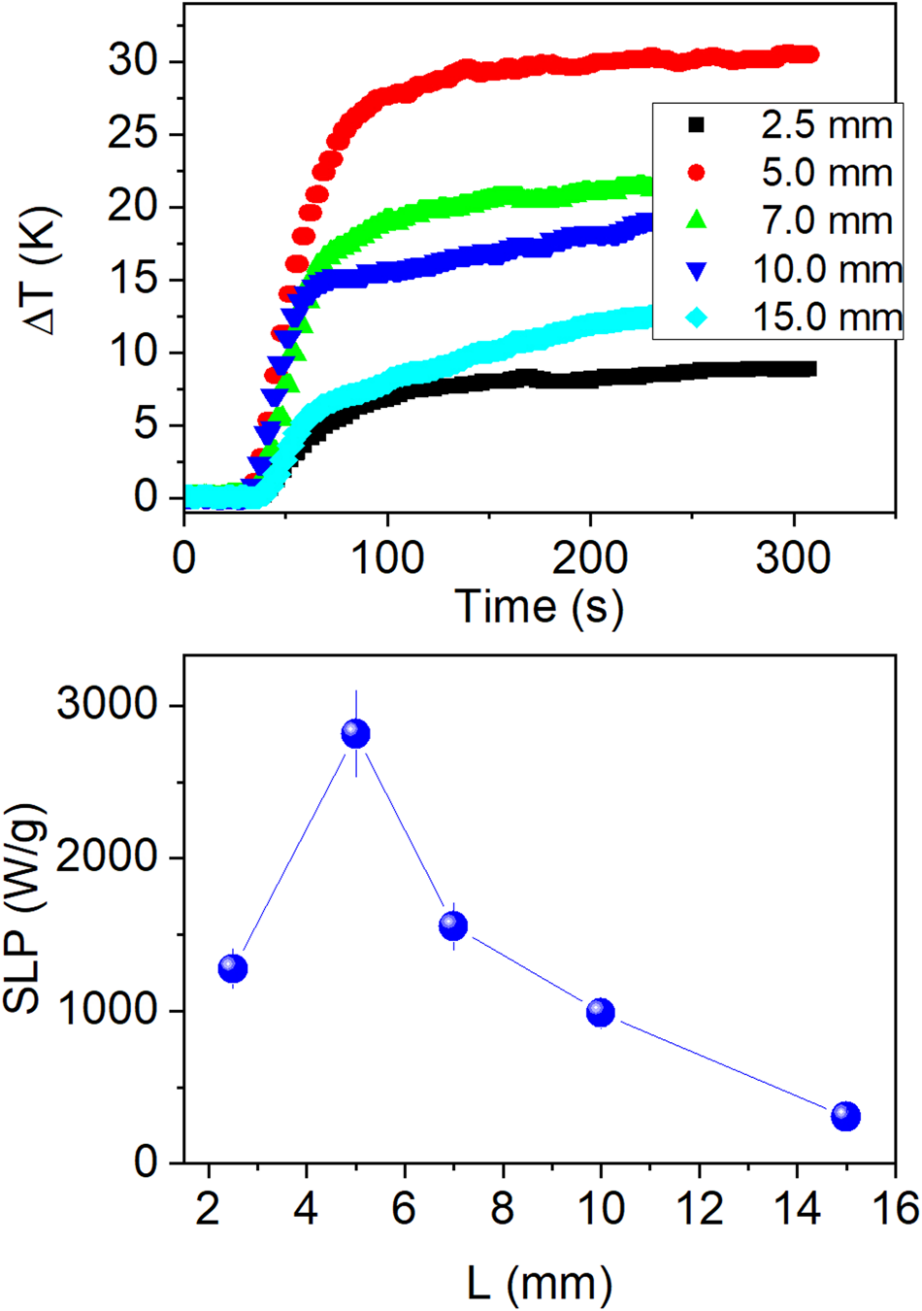
Heating curves (above) and SLP values (below) as a function of MWs length *L* measured for *n* = 20 at *f* = 625 kHz and *H* = 36 Oe. The SLP is calculated per unit of magnetic mass.

The effect of MWs length on *SLP* is shown in Fig. 8. As can be seen, the *SLP* almost doubles when the *L* varies from 2.5 to 5 mm and then decreases for longer lengths. The small value at 2.5 mm can be originated by the experimental setup of the MWs in water. Unlike magnetic colloids where nanoparticles are distributed in the whole volume producing a homogeneous temperature distribution, in the case of MWs they occupy only a part of the water volume and the mean temperature measured in water is due to the heat transferred from MWs by convection, conduction and irradiation to the water. The vial for the experiments has 1 cm high of water, and the 2.5 mm long MWs (1/4 of the total high) with a magnetic mass of 0.27 mg are located at the bottom of the vial. The water is a huge heat sink for the smallest MWs, and the heat dissipated by the MWs in the water could give an apparent result of a smaller heating efficiency for the 2.5 mm MWs. As the length increases to 5 mm, the magnetic mass increase and the *SLP* also increases.

However, for *L* > 5 mm, the *SLP* decreases again despite the magnetic mass increases. The wavelengths for frequencies from 600 to 100 kHz (used in this work) range from 0.5 to 3 km, therefore, the effect of a “characteristic length” of the MWs as the source of this decrease has to be discarded. The origin of this dependence can be understood by taking a look on the different hysteresis loops shapes, as shown in Fig. 3. It is clear that the highest susceptibility corresponds to the shorter lengths, and the susceptibility decreases as length increases. Furthermore, the area under the hysteresis loop and the magnetic susceptibility are higher for shorter length. Therefore, the origin of colossal *SLP* values hinges on the strong dependence of the susceptibility with the length of the negative magnetostrictive MWs.

#### MWs in air

The measurements in water allow determining the *SLP* of the samples; however, it is interesting to know if the number of MWs can affect the heating efficiency due to changes in the magnetostatic energy. For this purpose, the next experiments have been done in air and the data has been recorded with a thermographic camera, as reported in the Experimental Section.

**Figure 9:**
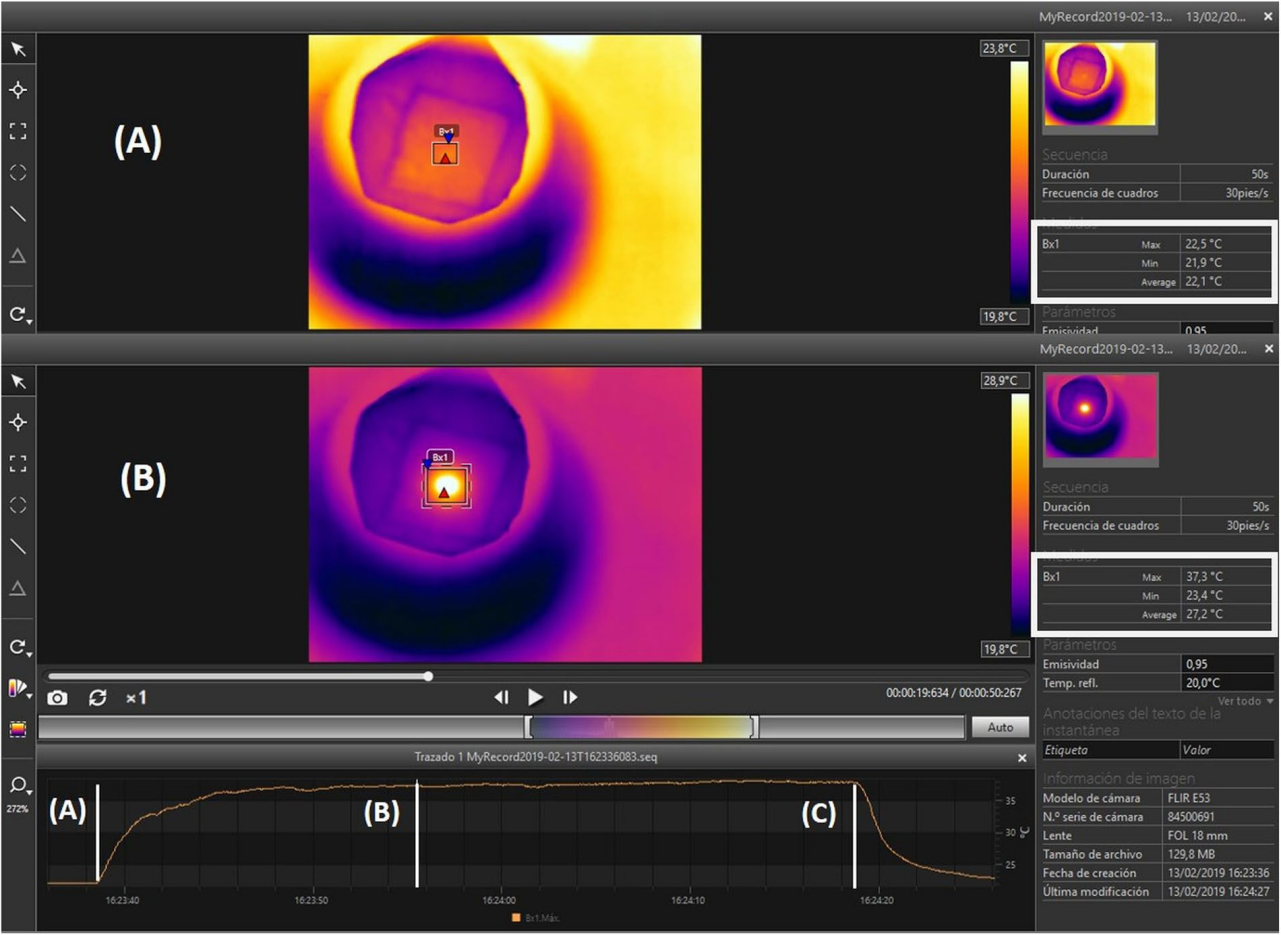
Heating images taken by IR camera for *n* = 2 with *L* = 5 mm separated by *d* < 0.5 mm. The field is *H* = 36 Oe at *f* = 625 kHz. (**A**) The field is off and the temperature measured at the MWs is 22.5 °C. (**B**) The temperature increases up to 37.7 °C in only 10 s. (**C**) The field is turned off and the temperature decreases.

Unfortunately, it is not possible to calculate the *SLP* of the MWs in air because: (1) heat capacity of MWs is unknown, (2) the air surrounding them has a four times smaller heat capacity (1.0 J/gK) than water (4.18 J/gK), it means that less energy is necessary to increase the temperature of air than of the water, but (3) the thermal conductivity of air (0.026 W/m·K) is much smaller than water (0.609 W/m·K), resulting in a significant thermal gradient during measurements, as can be seen in Fig. 9.

Even though the *SLP* cannot be calculated from the heating curves in air, the *SLP* for *n* = 20 and *L* = 5 mm is known in water and it is the same value for MWs in air because the *SLP* depends only on the intrinsic properties of the magnetic material and on the applied field but not on the surrounding media.

**Figure 10:**
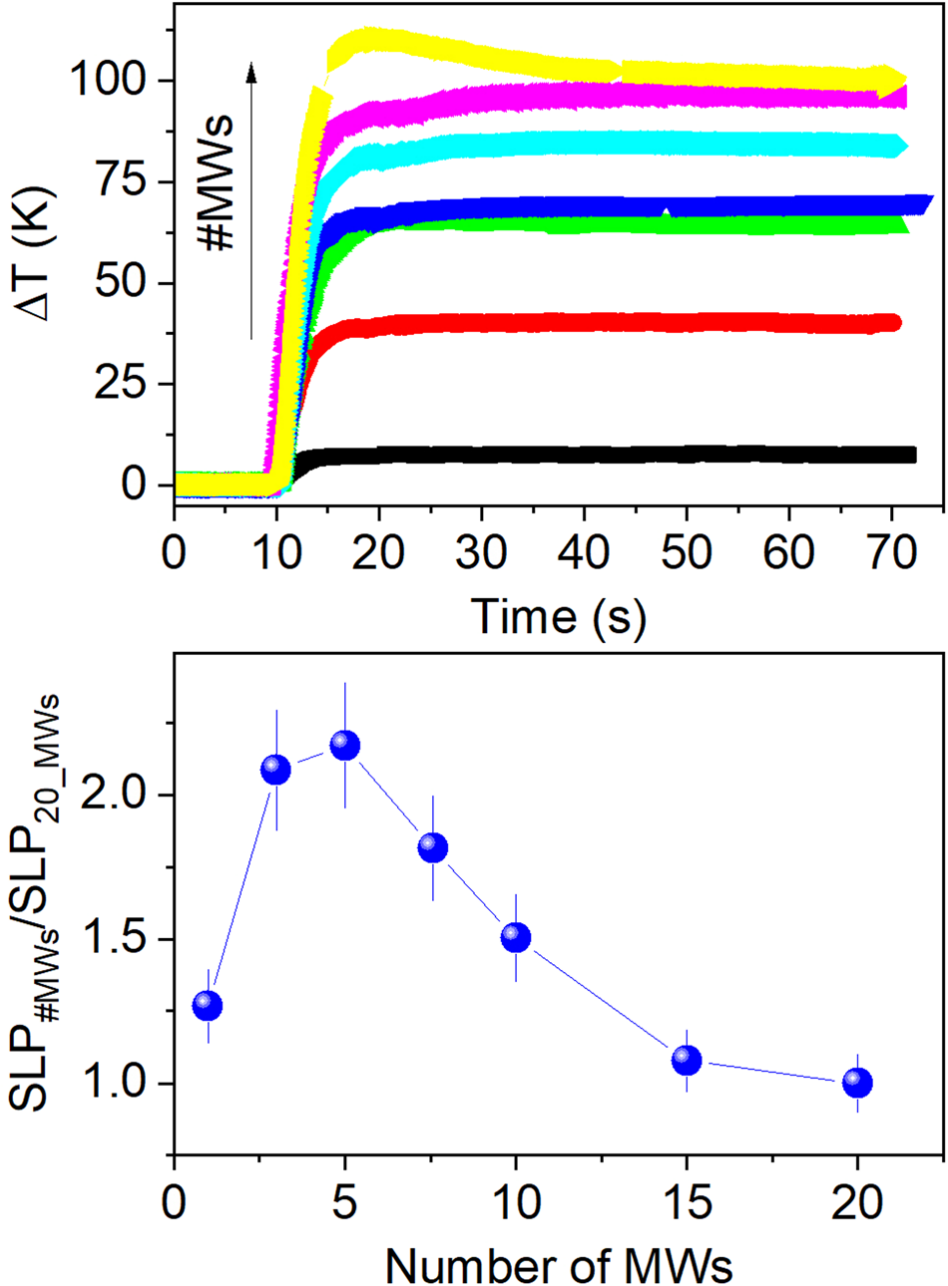
Heating curves (above) and temperature increase rate per magnetic mass as a function of the number of MWs in air (bellow) normalized to the value at *n* = 20. Length *L* = 5 mm, *H* = 36 Oe and *f* = 625 kHz. The arrow indicates the increasing number of MWs.

For a system of non-interacting magnetic nanoparticles, the *SLP* is independent of the magnetic mass^44^; therefore, in the case of non-interacting MWs, the temperature increase per unit mass should be the same for any numbers of MWs. Figure 10 shows the temperature increase per unit mass for any number of MWs (*n*) normalized to the value for 20 MWs, which is the same as in water. As the number of MWs decreases, this value increases, indicating that the heat released by the MWs is higher for a smaller number of MWs. It suggests that the interactions between MWs plays a significant role for the heat release. All measurements have been performed in air with *L* = 5 mm at *H* = 36 Oe and *f* = 625 kHz.

As it is well known, the dipolar interactions can improve or decrease the *SLP* depending on the magnetic properties of the materials and the applied field^44,45^. In the case of MWs, the dipolar interactions are often significant and can even give rise to hysteresis loops splitting in MWs with positive magnetostriction^46,47^. As discussed previously, the magnetic domain of the inner core are axial in short lengths and, in order to minimize the magnetostatic energy, there appears closure domains for *n* = 2, 3, 4, 5 and 6 MWs. The presence of closure domains increases the remanence up to *M*_*r*_ = 80%, i.e., it maximizes the area under the hysteresis cycles and increases the susceptibility, as well. For a larger number of MWs, the susceptibility and remanence decrease again, as shown in Fig. 4. This can be related to the heating curves, it is small for a single MW, it increases for *n* = 2, 3, 4, 5 and 6 MWs, and then decreases again for a larger number of MWs (Fig. 10). It is worth noting that the *M*_*r*_ decreases from 80% for *n* = 5 to 50% for *n* = 10 whereas the heating rate per mass diminishes in a similar proportion for these MWs (see Figs. 4 and 10), i.e., regarding the values for *n* = 5, the remanence and heating rate per mass for *n* = 10 are reduced to 62.5% and 69.1%, respectively. In a similar way, the mass susceptibility, χ_g_, are 11.5(5), 40.6(5) and 23.7(5) emu/g·Oe for 1, 5 and 20 MWs, respectively, i.e, the susceptibility first increases from 1 to 5 MWs and then decreases for a larger number of MWs, as already reported for other authors^48^. These results suggest that the magnetostatic interactions between MWs play the major role for the heating efficiency at radiofrequency fields.

Figure 9 shows the images and heating curve measured by the IR camera of 2 MWs separated by less than 0.5 mm and subjected to a field of *H*_*C*_ = 36 Oe at *f* = 625 kHz. The initial temperature is 22.5 °C and increases up to 37.7 °C in only 16 s. At this temperature, the system reaches the thermodynamic equilibrium with the environment and the temperature remains constant despite the field is still on. When the field is turned off, the temperature decreases as fast as it has increased.

**Figure 11:**
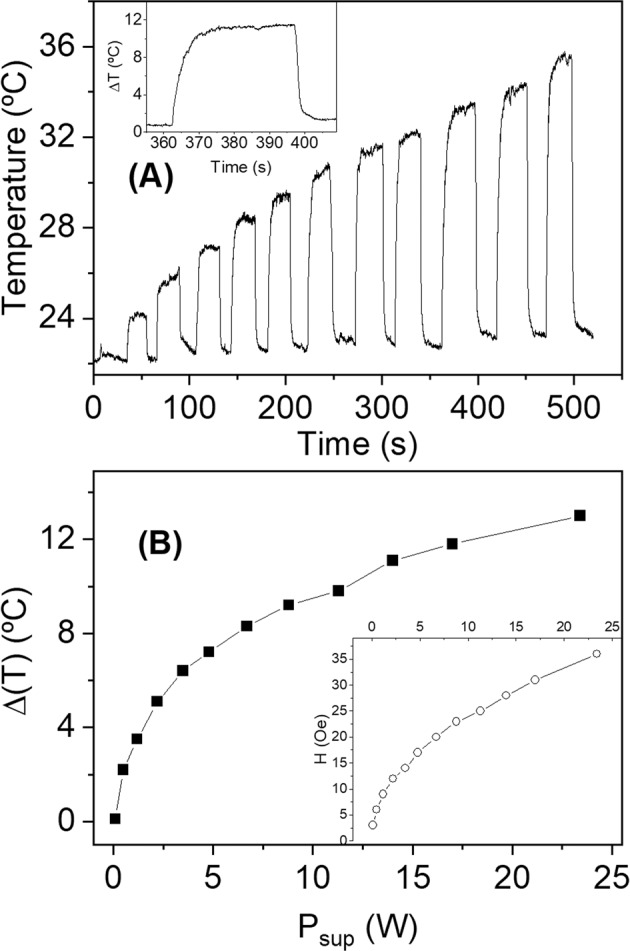
(**A**) Temperature of a single MW30 with turn-on and turn-off sequence at different field amplitudes and *f* = 625 kHz, the inset shows a detail of a heating up and cooling down process. (**B**) Temperature increase as a function of the power supplied by the equipment. (*n* = 1, *L* = 5 mm).

In order to demonstrate how efficient these MWs are, an experiment was performed in air with *n* = 1 and *L* = 5 mm. The temperature increase is measured with the infrared camera, and the power supplied by the equipment is calculated as ${P}_{\sup }=IV$, with *V* the applied voltage and *I* the current in the coil. From Fig. 11 and Table 1, it can be seen that the power supplied required for a temperature increase of 10 °C is only 12 W for a single MWs and, moreover, it takes only 5–10 s to reach the maximum temperature. If the power supplied increases twofold (*P*_sup_ = 24W), the temperature increases only up to 13 °C, i.e., the MWs are more efficient at relative low field (power supplied).

**Table 1:** Applied voltage *V*, current *I*, power supplied *V*, field amplitude *H* and temperature increase *ΔT* of a single MW with *L* = 5 mm in air. Temperature increase was measured with the infrared camera. Field frequency is *f* = 625 kHz.

*H* (Oe)	*V* (V)	*I* (A)	*P*_*sup*_ (W)	*ΔT* (°C)
3	1	0.10	0.1	0.1
6	2	0.25	0.5	2.2
9	3	0.40	1.2	3.5
12	4	0.55	2.2	5.1
14	5	0.70	3.5	6.4
17	6	0.80	4.8	7.2
20	7	0.95	6.7	8.3
23	8	1.10	8.8	9.2
25	9	1.25	11.3	9.8
28	10	1.40	14.0	11.1
31	11	1.55	17.1	11.8
36	13	1.80	23.4	13

By using *n* = 10 at *H* = 36 Oe and *f* = 625 kHz, the power supply is still 24 W, the heating efficiency decreases (see Fig. 10), but the temperature increase is *ΔT* ≈ 72 °C in only 15 s (see Movie S1 in Sup. Inf.).

A Cu wire with ~100 µm diameter was subjected to the same condition of field frequency and amplitude and no temperature increase was observed, confirming that the magnetization reversal plays the fundamental role in the heating mechanisms.

## Discussion

In the literature on magnetic particle hyperthermia there is no discussion about the power supplied by the source supply because the main objective is to kill as many cancer cells as possible regardless the energy spent in the process. As any other remote process, the energy supplied by the source is much higher than the energy converted into heating. However, if induction heating will be used for different catalysis process^7,11^, the power consume is a parameter that has to be considered.

In order to explain the name of “colossal”, a comparison between these MWs and standard iron oxide nanoparticles measured in the same equipment (Magnetherm 1.5 of Nanotherics) is shown. Considering the results for 14 nm γ-Fe_2_O_3_ measured at 100 Oe and 523 kHz by de la Presa *et al*.^49^ (see Fig. 5 of ref. ^49^) it is possible to evaluate the power supplied by the power source converted into heat by the nanoparticles and compare it with the present MWs. This ratio is defined as *r* = *P*_*s*_/*P*_*h*_, where *P*_*s*_ is the power supplied by the power source and *P*_*h*_ is the power loss released into heat by the magnetic materials. The Table 2 shows the values for power supplied, the heat release and the materials.

**Table 2:** Data of the materials (for γ-Fe_2_O_3_ see Fig. 5 of ref. ^49^), mass (*m*), frequency (*f*), field amplitude (*Oe*), voltage (*V*), current (*I*), power supplied by the power source (*P*_*s*_), *SLP*, heat realized by the magnetic nanoparticles (*P*_*h*_) calculated as the product of SLP by the magnetic mass, and the ratio *r* = *P*_*s*_/*P*_*h*_.

Materials	*m* (mg)	*f* (kHz)	*H* (Oe)	*V* (V)	*I* (A)	*P*_*s*_ = V · I(W)	*SLP* (W/g)	*P*_*h*_ = *SLP m* (W)	r = *P*_*s*_/*P*_*h*_ (%)
γ-Fe_2_O_3_ *d* = 13 nm	8.5	523	95	28.8	12.6	363	48.3	0.41	0.11
MWs *L* = 5 mm *n* = 20	0.54	625	36	13	1.8	24	2814	1.4	5.8

**Figure 12:**
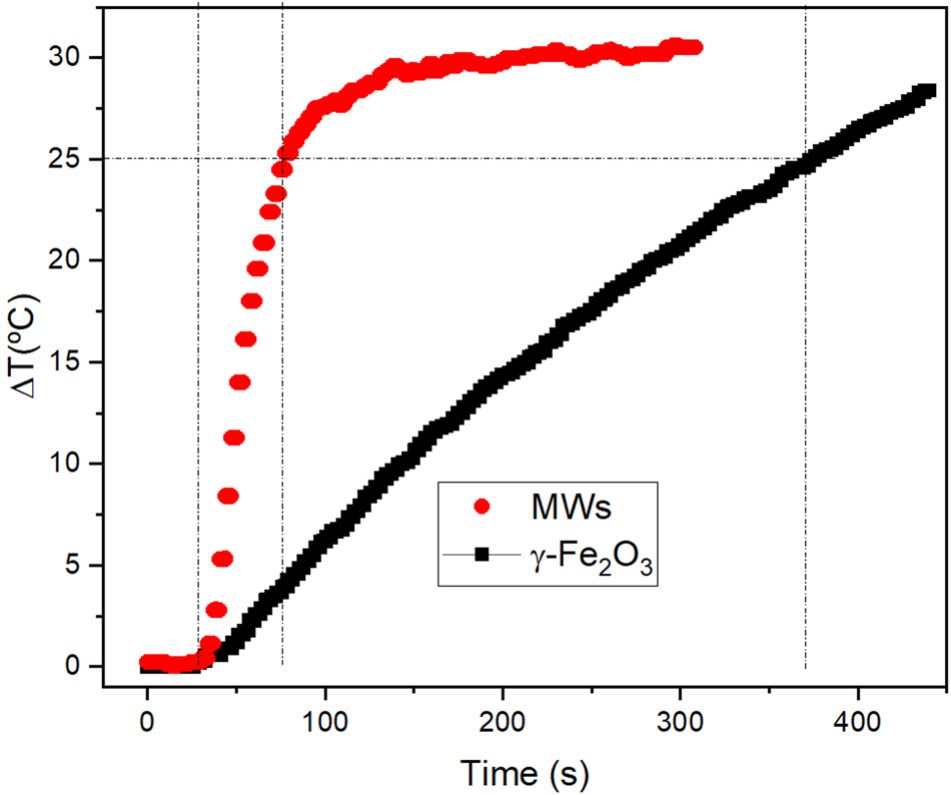
Temperature increase produced by 0.54 mg of MWs and 8.5 mg of γ-Fe_2_O_3_ when the power supplied by the source are 24 W and 363 W, respectively. (data on γ-Fe_2_O_3_ has been reprinted with permission from J. Phys. Chem C 2012, 116, 25602. Copyright 2012 American Chemical Society).

As can be seen from Table 2, for a MW mass which is 15 times smaller than the nanoparticles mass, the ratio *r* is ~50 times higher. To illustrate this effect, Fig. 12 shows two heating curves, one corresponding to *m* = 0.54 mg of the MWs and the other one to *m* = 8.5 mg of the γ-Fe_2_O_3_. Whereas the MWs can increase the temperature of water by 25 °C in less than 1 min, the γ-Fe_2_O_3_ nanoparticles need more than 5 min to reach the same temperature, despite of having 15 times more mass and 15 times more power supplied by the source.

The size of the of MWs make that the heating can be originated by two different processes: the hysteresis losses and eddy-current losses. In the case of eddy currents, the power loss increases proportionally to the square root of the frequency according to: ${P}_{eddy}=\sqrt{\mu pf}{H}_{0}^{2}$, where *P*_*eddy*_ is the power loss per unit mass, *μ* is the effective permeability, *ρ* the resistivity, and *f* the frequency of the field^14^. On the other hand, according to the Linear Response Theory, the magnetic hysteresis power loss is given by ${P}_{hyst}=2\mu \pi \chi ^{\prime\prime} (t)f{H}_{0}^{2}$, where *P*_*hyst*_ is the power loss density and *χ*″*(t)* is the imaginary part of the complex susceptibility $\tilde{\chi }(t)$^50^. Both power losses depend on the square of the field amplitude, but they differ in the frequency field dependence. In the eddy-current mechanism, the power loss depends on the square root of the field frequency whereas for magnetic hysteresis loss it varies linearly with the field frequency. This should be the way to distinguish both processes. However, as can be seen in Fig. 7, the frequency dependence is practically linear and the amplitude dependence is not quadratic; therefore, the processes involved in the heating are complex and cannot be explained with any of these two simple equations.

In order to determine which is the contribution of the hysteresis losses, a first approximation of the maximum contribution of the hysteresis losses to the heating can be done. Let’s assume that a MW30 with *L* = 5 mm has a perfectly square hysteresis loop with the *M*_*s*_ and *H*_*c*_ values measured at *f* = 50 Hz, and these values are still valid at *f* = 625 kHz, this would be then the maximum possible area under the hysteresis cycle. In the case of nanoparticles, remanence and coercivity can be much smaller at high frequencies than at low frequencies^45^, and, additionally, depending on relation between magnetic and thermal energy, the susceptibility can vary with the frequency. Unlike nanoparticles, the coercivity in MWs can increase at high frequencies because the induced current creates a field that opposes to the applied field, giving place to an effective coercive field higher than at low frequencies. Considering *M*_*s*_ = 100 emu/g, *H*_*C*_ = 5 Oe and *f* = 625 kHz, an approximated value of the heating loss is $SLP=fA=30W/g$, where *A* is the area of the hysteresis loop and *f* the field frequency. Thus, hypothetical hysteresis loss is at least 100 times smaller than the measured one. It can be concluded, that, even when hysteresis losses could contribute to the heating, they cannot explain the colossal heating efficiency of these MWs.

The induced magnetic field inside the NWs always opposes to the change of magnetization, especially at high frequency, and the eddy current losses can be significant^51^. As frequency increases, the eddy-current is damped away and the effective resistance increases because the effective area decreases, consequently, this skin effect causes a heating increase. The *SLP*_*eddy*_ depends on how the susceptibility can change with the field amplitude and frequency. The fact that the *SLP* at *f* = 331 kHz increases even at 120 Oe (a field much higher than the anisotropy field) suggests that the maximum susceptibility has not been reached at this frequency.

Both, Co-rich MWs with nearly zero magnetostriction and Fe-rich MWs with positive magnetostriction, are able to release heat under radiofrequency fields; however, our results show that the Co-rich MWs present a colossal heating efficiency. The magnetic domains become axial for a 5 mm Co-reach MWs similarly to the Fe-rich MWs^14^, but with higher coercivity. The main difference between both kinds of MWs probably lies in the concentric closure domains, which are circular for the Co-rich (see Fig. 3) but radial for the Fe-rich MWs. As previously discussed, the hysteresis losses play a minor role in the heating efficiency, and the eddy-current loss, which is proportional to the susceptibility, the major one. These concentric domains are responsible for the GMI in Co-rich MWs^52^, because the magnetization of the outer shell can reverse with the applied field even at the microwaves frequencies. These concentric closure domains could be responsible for the colossal heating due to the susceptibility enhancement at high frequencies; furthermore, it is unlikely that the axial domain of the inner core can reverse at these frequencies. Therefore, MWs with nearly zero magnetostriction are the key candidates for colossal heating at radiofrequency fields.

The heating and cooling sequence shown in Fig. 11 demonstrates the potential applications of these MWs beyond the hyperthermia cancer treatments. These MWs could be used in applications which need a fast heating by a contact less system, for example, to prevent the freezing in the transport systems or to induce a catalytic reaction.

## Conclusion

Co-rich MWs with nearly zero magnetostriction has been proven to have colossal heating efficiency under radiofrequency field. The *SLP* value for set of 20 MWs is around 2800 W/g, but, what it is really surprising, is the low field required to reach such huge value: only 36 Oe at 625 kHz. This colossal efficiency is conditional upon the MWs length and number. The MWs length plays a significant role since length reduction makes the magnetic domains change from radial to axial domain, as deduced from the hysteresis curve shapes. As a consequence, a length reduction from 15 to 5 mm gives place to a *SLP* increase from 300 to 2800 W/g, showing the relevance of the domains ordering. The number of MWs sets side by side is also a significant parameter because magnetostatic interactions decreases the remanence (and the susceptibility) from *M*_*r*_ = 80% for *n* = 2–6 to *M*_*r*_ = 50% for *n* = 10 suggesting the formation of closure domains that diminish the magnetostatic energy. This change of hysteresis cycle shape affects also the heating efficiency, which decreases for *n* = 10 by a factor of 2/3 regarding *n* = 5.

It is assumed that the origin of this colossal heating lies in the eddy-currents, even though a small hysteresis losses contribution cannot be discarded at all. The eddy currents are generated by electromagnetic induction $B=M+\chi H$ and lead to Joule heating of the material. Unlike non-magnetic metals, the eddy–currents in Co-rich MWs are enhanced by the magnetization reversal and, thus, the magnetic susceptibility at radiofrequency range becomes a key factor for the heating efficiency. Since the magnetic domains of Co-rich MWs with *L* = 5 mm becomes axial, the circumferential domains play probable the main role in the susceptibility at high frequency. Therefore, short MWs with nearly magnetostriction are key candidates to show colossal heating.

Finally, it is shown that this colossal heating efficiency favours the design of induction heating dispositive with very low energy consuming, a unique MW can increase the temperature by 10 °C in 5 s with only 12 W.

## Experimental Section

The materials used for these experiments are soft magnetic Fe_2.25_Co_72.75_Si_10_B_15_ (Co-rich MWs) consisting of an amorphous metallic core coated by a Pyrex shell fabricated by means of the modified Taylor-Ulitovsky method. The external diameter *D* = 49.4 μm with magnetic diameter *d*_*m*_ = 31.4 μm (MW30) and total mass *m* = 0.08 mg/cm, with 67.5% of the mass corresponding to the magnetic core. Additionally, as-cast and thermal treated MWs at 300 °C were studied in order to investigate the effect of annealing on heating efficiency. MWs with different lengths *L* ranging from 2.5 to 80 mm were studied as well different numbers of MWs with *L* = 5 mm. Besides, a 5 mm Cu wire with 100 μm diameter was measured at the same fields in order to investigate the effects of eddy-currents in a non-magnetic material at these field frequencies and amplitudes.

Hysteresis loops of the as-cast and annealed at 300 °C MW30 were measured at *f* = 50 Hz with a maximum applied field of *H* = 30 Oe. Besides, the hysteresis loops have been measured for different lengths (*L* = 5, 15 and 80 mm) and for different numbers of MW30, from *n* = 1 to 20.

The MWs were subjected to radiofrequency fields in the commercial system Magnetherm 1.5 (Nanotherics). A 17 turns-coil combined with 5 different capacitors allowed for 5 resonant frequencies: 110, 160, 330, 450 and 625 kHz with a maximum applied field of 200 Oe, which permits the investigation of the effect of magnetic field frequency and amplitude on the heating efficiency. The coil temperature has been controlled through a closed circuit of water maintained at 16 °C with a cryostat bath.

For the experiments in water, the temperature has been measured with a fiberoptical thermometer and registered with a computer. For the experiments in air, the temperature has been recorded with a thermographic camera FLIR E53, field of vision 24° × 18° Lens, and 240 × 180 pixels resolution. This camera allows the simultaneous measurement of different points; in particular, the MWs and the coil have been measured simultaneously in order to characterize the thermal contribution of the coil. The program FLIR Tools® has been used for video record and data acquisition.

## Supplementary information

Supplementary Information.

Video S1.
