# Supplementary material for: Colossal heating efficiency via eddy currents in amorphous microwires with nearly zero magnetostriction
Source: Sci Rep. 2020 Jan 17;10:602. doi: 10.1038/s41598-020-57434-8 (PMC6969244; doi:10.1038/s41598-020-57434-8)
Supplement: Supplementary file 1 — Supplementary Information. [file 41598_2020_57434_MOESM1_ESM.docx]

Colossal heating efficiency via eddy currents in amorphous microwires with nearly zero magnetostriction.

Irene Morales,^a,†^ Diego Archilla,^a,†^ Patricia de la Presa,^a,b,*^ Antonio Hernando,^a,b,c^ Pilar Marin^a,b^

^(a)^Instituto de Magnetismo Aplicado, UCM-ADIF-CSIC, A6 22,500 Km, 20230 Las Rozas, Spain

^(b)^Departamento Física de Materiales, Univ. Complutense de Madrid, 28040 Madrid, Spain

^(c)^ Donostia International Physics Center DIPC, Paseo Manuel de Lardizabal 4, 2018 Donostia-San Sebastián, Spain and IMDEA Nanociencia, Faraday, 9, 28049 Madrid, Spain

^†^ Both authors contributed equally to this article.

* Correspondence and requests for materials should be addressed to PP ([pmpresa@ucm.es](mailto:pmpresa@ucm.es))

**Video S1**: The video shows the temperature increase (upper left corner) for *n*  = 10 and *L* = 5 mm when a radiofrequency field with *H* = 36 Oe and *f* =625 kHz is applied. The field is turned on at *t* = 1 s, and turned off at t = 16 s. The whole temperature increase is *ΔT* =71.7 ºC
